# Pyramiding of transgenic immune receptors from primary and tertiary wheat gene pools improves powdery mildew resistance in the field

**DOI:** 10.1093/jxb/erad493

**Published:** 2023-12-10

**Authors:** Teresa Koller, Marcela Camenzind, , Esther Jung, Susanne Brunner, Gerhard Herren, Cygni Armbruster, Beat Keller

**Affiliations:** Department of Plant and Microbial Biology, University of Zurich, Zollikerstrasse 107, 8008 Zurich, Switzerland; Department of Plant and Microbial Biology, University of Zurich, Zollikerstrasse 107, 8008 Zurich, Switzerland; Department of Plant and Microbial Biology, University of Zurich, Zollikerstrasse 107, 8008 Zurich, Switzerland; Agroscope, Reckenholzstrasse 191, 8046 Zurich, Switzerland; Department of Plant and Microbial Biology, University of Zurich, Zollikerstrasse 107, 8008 Zurich, Switzerland; Department of Plant and Microbial Biology, University of Zurich, Zollikerstrasse 107, 8008 Zurich, Switzerland; Department of Plant and Microbial Biology, University of Zurich, Zollikerstrasse 107, 8008 Zurich, Switzerland; CIMMYT, Mexico

**Keywords:** Disease resistance, field trial, *Pm17*, powdery mildew, tertiary gene pool, transgenic crops, wheat

## Abstract

Introgression of resistance genes from wild or related species is a common strategy to improve disease resistance of wheat cultivars. *Pm17* is a gene that confers powdery mildew resistance in wheat. It encodes an NLR type of immune receptor and was introgressed from rye to wheat as part of the 1RS chromosome arm translocation several decades ago. So far it has not been possible to separate *Pm17* from its co-introgressed rye genes due to suppressed recombination. Here we tested in the field transgenic Bobwhite wheat overexpressing *Pm17* without any other rye genes. Four transgenic events showed high levels of PM17 protein accumulation, strong powdery mildew resistance, and no pleiotropic effects during three field seasons. We used a combined approach of transgene insertion and cross-breeding to generate lines co-expressing *Pm17* and *Pm3*, or *Pm17* and *Pm8*. *Blumeria graminis* f. sp. *tritici* infection tests confirmed additive, race-specific resistance of the two pyramided transgenes in lines Pm17+Pm3b and Pm17+Pm8. Furthermore, pyramided lines showed strong powdery mildew resistance during three field seasons. We conclude that the combination of overexpressed *NLR* genes from the extended gene pool broadens and diversifies wheat disease resistance.

## Teaser

Powdery mildew resistance genes from the primary and tertiary gene pools of wheat were overexpressed and combined in wheat cultivar Bobwhite. Transgenic plants showed additive race-specific and field resistance.

## Introduction

Wheat (*Triticum aestivum*) is a major staple crop for human consumption and animal feed production. Protecting wheat from diseases is crucial for global food security. Disease resistance breeding is one of the major contributors to preventing crop loss. The identification and informed deployment of disease resistance genes is the basis of sustainable resistance breeding and resistance management. Powdery mildew is an important wheat disease caused by the biotrophic fungus *Blumeria graminis* f. sp. *tritici* (*Bgt*). In conditions favoring *Bgt* growth and propagation, yield losses are in the range of 5–15%, but in severe *Bgt* epidemics, higher yield losses have been reported (reviewed in Singh *et al.*, 2016). In *Triticeae* species, several powdery mildew resistance genes encode nucleotide-binding leucine-rich repeat receptor (NLR) proteins. In addition, a few non-NLR types of immune receptors conferring powdery mildew resistance have been identified in wheat (Sánchez-Martín and Keller, 2021). NLR-type resistance proteins recognize race-specific *Bgt* effectors and trigger a strong immune response (Bourras *et al.*, 2015, 2019; Sánchez-Martín *et al.*, 2016; Wang *et al.*, 2023). Resistance mechanisms of non-NLR types of powdery mildew resistance genes such as *Pm4* and *Pm24* are largely unknown (Lu *et al.*, 2020; Sánchez-Martín *et al.*, 2021). It is important to understand the molecular function of disease resistance proteins and later to test the concepts developed for resistance improvement in an actual field setting, to optimally deploy disease resistance genes in resistance breeding programs and later in agriculture. In particular the NLR type of immunity is prone to break down after a few years, when the same NLR-encoding resistance gene is deployed as the only active resistance gene in cultivars widely grown in space and time, because pathogen populations evolve to delete or mutate recognized effectors (Jones and Dangl, 2006; Ngou *et al.*, 2022). One strategy to extend the durability of the highly effective NLRs is the combination or stacking, also called pyramiding, of several *NLR*-encoding genes in elite cultivars (McDonald and Linde, 2002).

In previous studies we overexpressed alleles of the powdery mildew resistance gene *Pm3* in spring wheat cultivar Bobwhite, by using the maize ubiquitin (*ubi*) promoter, and tested the transgenic lines in the field (Brunner *et al.*, 2011, 2012; Koller *et al.*, 2019). Furthermore, we pyramided several combinations of two *Pm3* alleles by crossing transgenic Bobwhite lines overexpressing single *Pm3* alleles (Stirnweis *et al.*, 2014). Pyramided lines were tested in the field and showed improved powdery mildew resistance (Koller *et al.*, 2018). The improved powdery mildew resistance in the field was attributed to the two effects of enhanced total *Pm3* transgene expression levels and allele specificity combinations that act additively (Koller *et al.*, 2018). Similar studies were performed in transgenic potato where pyramided late blight resistance genes showed improved resistance in the field (Jo *et al.*, 2014). The resistance genes originated from the extended gene pool of potato, namely from the crossable species *Solanum stoloniferum* and *Solanum venturii* (Jo *et al.*, 2014).

Wheat breeders use disease resistance genes from the primary, secondary, and tertiary gene pools of wheat to enhance elite cultivars (Walkowiak *et al.*, 2020). Deployment of genes from the tertiary gene pool is often hindered by linkage drag and poor outcome of crosses, and in the case of resistance genes, by genetic suppression (Chaudhary *et al.*, 2014). In this study we focused on the deployment of the *NLR*-encoding powdery mildew resistance gene *Pm17* from the tertiary gene pool of wheat. *Pm17* originates from the short arm of chromosome 1 of rye (1RS) and is localized on the 1RS.1AL translocation in wheat (Singh *et al.*, 2018) (Supplementary Fig. S1). *Pm17* is an ortholog of the wheat powdery mildew resistance gene *Pm3*, which is localized on 1AS (Yahiaoui *et al.*, 2006; Singh *et al.*, 2018) (Supplementary Fig. S1). Interestingly, there is a third ortholog of *Pm17* and *Pm3*, called *Pm8* (Hurni *et al.*, 2013). *Pm8* is localized on 1RS of 1RS.1BL and also originates from rye (Supplementary Fig. S1). *Pm8* provides powdery mildew resistance, but the resistance has broken down in many wheat growing regions worldwide due to adaptation of the recognized AVRPM8 effector (Kunz *et al.*, 2023). As the case of *Pm17* and *Pm8* shows, 1RS from rye was introgressed into wheat several times and integrated into the wheat genome on the short arm of either chromosome 1A or chromosome 1B (Schlegel and Korzun, 1997) (Supplementary Fig. S1). Repressed recombination on 1RS of wheat chromosomes 1RS.1BL and 1RS.1AL makes it difficult to study the effects of single 1RS-localized genes. For example, the contribution of *Pm17* to powdery mildew resistance of wheat cultivar Amigo is masked by the presence of a second *Pm* resistance gene on 1RS.1AL (Müller *et al.*, 2022). Transgenic wheat events overexpressing *Pm17* and *Pm8* as single transgenes were generated using the powdery mildew susceptible spring wheat cultivar Bobwhite (Hurni *et al.*, 2013; Singh *et al.*, 2018). Infection tests on these transgenic Bobwhite events confirmed the race-specific powdery mildew resistance function of the *Pm17* and *Pm8* transgenes (Hurni *et al.*, 2013; Singh *et al.*, 2018). Furthermore, Hurni *et al.*, 2014 showed that *Pm8* is suppressed by the wheat *Pm3* gene. Resistance genes from the tertiary gene pool are prone to being suppressed by endogenous resistance genes (Chaudhary *et al.*, 2014).

To elucidate the race-specific powdery mildew resistance mechanisms of *Pm3*, *Pm17*, and *Pm8*, the corresponding avirulence effector genes (*Avrs*) from *Bgt* were previously identified and named *AvrPm3a*, *AvrPm3b*, *AvrPm3d*, *AvrPm8*, and *AvrPm17* (Bourras *et al.*, 2015, 2019; Müller *et al.*, 2022; Kunz *et al.*, 2023). These *Avr* genes encode the recognized avirulence effector proteins AVRPM3A, AVRPM3B, AVRPM3D, AVRPM8, and AVRPM17. Furthermore, a *Bgt* suppressor named *SvrPm3* was identified (Bourras *et al.*, 2015; Parlange *et al.*, 2015). *SvrPm3* suppresses *Pm3*–*AvrPm3* immune signaling (Bourras *et al.*, 2015, 2019).

Here we tested the powdery mildew resistance profile of transgenic Bobwhite wheat plants overexpressing single *Pm17* or single *Pm8*, and combinations of two overexpressed transgenes *Pm17* and *Pm3*, or *Pm17* and *Pm8*. We performed both seedling assays using *Bgt* isolates with specific *Avr*-gene combinations and field trials where plants are exposed to the natural, local powdery mildew population. This approach allowed us to evaluate the powdery mildew resistance profile of overexpressed and combined powdery mildew resistance genes *Pm17* and *Pm8* in wheat, in the absence of co-segregating genes from rye.

## Materials and methods

### Transgenic wheat lines

*Pm17* transgenic events Pm17#110, Pm17#122, Pm17#34, and Pm17#181 and the corresponding sister lines were previously generated and described by (Singh *et al.*, 2018). *Pm8* transgenic events Pm8#12, Pm8#59, and sister line Pm8#59-sis were previously generated and described by Hurni *et al.* (2013). Transgenic events Pm3b#64 and Pm3CS#19 were generated using the same spring wheat cultivar Bobwhite SH 98 26, the same plasmid backbones containing the maize ubiquitin (*ubi*) promoter for transgene overexpression, the same selection marker construct expressing *manA*, and the same protocols as for the generation of the *Pm17* and *Pm8* events (Hurni *et al.*, 2013; Singh *et al.*, 2018). The *Pm17*, *Pm3b*, and *Pm3CS* transgenes were C-terminally fused to a sequence that encodes a hemagglutinin (HA)-tag for protein detection. The *Pm8* transgenes were C-terminally fused to a sequence that encodes a c-myc tag (called myc) for protein detection. Pyramided line Pm17+Pm3b was generated by cross-breeding of parental events Pm17#110 and Pm3b#64. Pyramided line Pm17+Pm3CS was generated by cross-breeding of parental events Pm17#110 and Pm3CS#19. Pyramided line Pm17+Pm8 was generated by cross-breeding of parental events Pm17#110 and Pm8#59. After the initial crossing, five generations were generated in the greenhouse and genotyped to select the final three pyramided lines Pm17+Pm3b, Pm17+Pm3CS, and Pm17+Pm8, which are all homozygous for both transgenes, *Pm17-HA* and *Pm3b-HA*, *Pm17-HA* and *Pm3CS-HA*, and *Pm17-HA* and *Pm8-myc*, respectively.

### Field trial set-up and scoring

Legal permits for field experiments with genetically modified plants were obtained prior to the field trials by the Federal Office for the Environment (permit #B18001). Field trials were carried out during years 2020 (field season 1), 2021 (field season 2), and 2022 (field season 3) at the so called ‘protected site’ (www.protectedsite.ch), an experimental field site for research trials with transgenic crops, which is located at Agroscope in Zurich Reckenholz (Romeis *et al.*, 2013; Brunner *et al.*, 2021). Wheat genotypes were grown in test plots of 1.5 m×1.0 m. Four test plots per genotype were grown in a randomized complete block design. Test plots were flanked by infection rows consisting of the powdery mildew susceptible wheat breeding line FAL94632 and cultivar Kanzler. Pots with susceptible wheat plants pre-infected in the greenhouse with Swiss powdery mildew isolate *Bgt* 96224 were planted into the infection rows as described by Koller *et al.* (2018). Powdery mildew scoring was performed as described by Brunner *et al.* (2011). Flag leaf chlorophyll content was measured using a portable chlorophyll meter (SPAD 502; Minolta, Osaka, Japan).

### Swiss powdery mildew isolate *Bgt* 96224

The high-quality reference genome sequence of *Bgt* 96224 (Wicker *et al.*, 2013; Müller *et al.*, 2019) showed that *Bgt* 96224 carries a copy of variant A of *AvrPm3b*, which encodes an effector recognized by wheat NLR PM3B (Bourras *et al.*, 2019). *Bgt* 96224 does not carry a functional copy of the suppressor gene *SvrPm3*. In wheat seedling assays, *Bgt* 96224 was avirulent on transgenic Bobwhite event Pm3b#64 overexpressing *Pm3b* (Supplementary Fig. S2). *Bgt* 96224 carries a copy of *AvrPm8*^*F43Y*^. Mutation F43Y results in an AVRPM8 variant not recognized by wheat NLR PM8 (Kunz *et al.*, 2023). *Bgt* 96224 is virulent on transgenic Bobwhite event Pm8#59 overexpressing *Pm8* (Supplementary Fig. S2). *Bgt* 96224 carries two copies of variant B of *AvrPm17*. AVRPM17 variant B triggers a hypersensitive response in the presence of wheat NLR PM17, but the response is not as strong as the one triggered by AVRPM17 variant A (Müller *et al.*, 2022). In seedling assays, *Bgt* 96224 was intermediately virulent (i.e. it grows slowly) on transgenic Bobwhite event Pm17#110 overexpressing *Pm17* (Supplementary Fig. S2).

### Field-grown flag leaf sample collection for RNA and protein extraction

For each wheat genotype, plants from four plots were sampled. Per plot, flag leaf samples of three plants were pooled. Fully developed flag leaves were cut at the leaf base, then the first 4 cm was discarded, before cutting a 1 cm long segment for RNA sampling and another 1 cm long segment for protein sampling. The three segments per plot were pooled in a tube and instantly frozen in dry ice. Samples were kept at −80 °C for storage.

### Reverse transcription–quantitative polymerase chain reaction

The frozen flag leaf samples collected in the field were weighed and ground with a Geno/Grinder®. RNA extraction was performed using the Dynabeads mRNA DIRECT Purification Kit (Thermo Fisher Scientific). Lysis binding buffer (LBB) 125 μl per 10 mg plant material was added to each sample. For reverse transcription of the eluted mRNA to cDNA, the Maxima H Minus cDNA Synthesis Master Mix Kit (Thermo Fisher Scientific) was used. As a control reaction (RT minus control), a few samples were treated with the DNase digest and reverse transcription step, but without the reverse transcriptase (Maxima H Minus RT). To analyse transgene expression, reverse transcription–quantitative polymerase chain reaction (RT-qPCR) was performed using the KAPA SYBR FAST qPCR Kit (Kapa Biosystems) on the CFX96 Real-Time PCR Detection System (Bio-Rad Laboratories). The reactions were run in technical duplicates of four biological replicates per wheat line. No template control (NTC) reactions were performed using water instead of cDNA. *ADP ribosylation factor* (*ADPRF*) was used as the reference gene against which the mRNA expression levels of the target genes were normalized (Giménez *et al.*, 2011). Thermocycling conditions for *manA* and *ADPRF* were 95 °C for 1 min, followed by 39 cycles of 95 °C for 3 s and then 60 °C for 20 s. For *Pm17* thermocycling conditions were 95 °C for 1 min, followed by 39 cycles of 95 °C for 3 s and then 63 °C for 20 s. The following primers (5ʹ–3ʹ sequences) were used: *Pm17*: GCCCGGTATGAAGTAACAGC and AGTTCCTTGGCTTCTCGACT; *manA:* GGAAGTGATGGCAAACTCCG and TTCTGCACCTTGTTTCACCG; reference gene *ADPRF*: TCTCATGGTTGGTCTCGATG and GGATGGTGGTGACGATCTCT. Primer efficiencies were analysed by creating a standard curve of a 1: 4 serial dilution and calculating the efficiencies using the CFX Maestro software (Bio-Rad Laboratories). Data were also analysed using the CFX Maestro software (Bio-Rad Laboratories) and graphs were created using R Studio (R version 4.1.3). Statistical analysis was performed using the Tukey’s honestly significant difference test (95% confidence interval) in R Studio (agricolae package v1.3.5; de Mendiburu, 2021).

### Protein detection

Proteins from frozen flag leaf samples were extracted using 500 μl protein extraction buffer (15 mM NaCl, 5 mM Tris–HCl pH 7.5, 0.5% Triton X-100; one tablet cOmplete^TM^ EDTA-free protease inhibitor cocktail (Roche) was added per 25 ml buffer). The total protein concentration of the extract was determined using the Pierce BCA Protein Assay Kit (Thermo Fisher Scientific). All protein samples were adjusted to the same concentration using 1× Laemmli buffer prior to loading. For SDS-PAGE, 8% SDS polyacrylamide separation gels were used. Fifteen microliters of protein sample were loaded per well and 5 μl of the PageRuler Plus Prestained Protein ladder (Thermo Fisher Scientific) as a protein size marker. After separation by gel electrophoresis at 100 V for 90 min, the proteins were transferred to a methanol-activated polyvinylidene difluoride membrane (Immobilon-P Transfer Membrane, Millipore) by wet transfer. The membranes were blocked with 5% non-fat milk powder in 1× Tris-buffered saline–Tween 20 (TBST) buffer for at least 45 min at room temperature. Detection of PM17–HA, PM3b–HA, and PM3CS–HA proteins was performed by incubating the membrane in a 1:1000 dilution of the horseradish peroxidase (HRP)-conjugated antibody (anti-HA–HRP, rat monoclonal, clone 3F10, Roche) for 1 h at room temperature. After incubation, the membrane was washed twice for 5 min and finally once for 10 min with 1× TBST. The WesternBright ECL HRP substrate (Advansta) was used for imaging of the peroxidase chemiluminescence. The signal was detected using the Fusion FX Imaging System together with Evolution Capture software in the chemiluminescence setting. Detection of myc-tagged PM8 protein was achieved as for HA-tagged proteins except for the following specific changes: after blocking, membranes were incubated with the peroxidase-conjugated c-Myc antibody GTX19312 from LubioScience (GeneTex) in a 1:3000 dilution for 90 min at room temperature. Next, membranes were washed twice for 5 min and twice for 10 min in 1× TBST before signal detection.

### *Bgt* infection test

*Blumeria graminis* f. sp. *tritici* (*Bgt*) isolates used in this work were obtained from our group’s powdery mildew collection. The powdery mildew isolates were propagated on the susceptible bread wheat cultivar Kanzler, essentially as described in Hurni *et al.* (2013). Briefly, segments of the first leaf from 12-day-old Kanzler plants were placed with the adaxial side up on plates with 0.5% agar and 0.24 mM benzimidazole (Parlange *et al.*, 2011). Leaf segments were then infected with the fungal spores and incubated at 20 °C for 7 d with 16 h of light per day to allow for sufficient colony growth. Phenotyping experiments were performed by placing 3 cm-long first leaf segments of the wheat lines of interest on benzimidazole agar plates. The powdery mildew susceptible wheat cultivar Kanzler was used as control. The leaf segments were infected with the fungal spores collected from propagation plates by dusting them homogeneously over the plates with a single-use glass pipette. The infected plates were incubated at 20 °C with 16 h of light per day. Photographs of the plates were taken 7 d after infection and virulence was scored by estimating the percentage of leaf coverage with fungal colonies (LC). The virulence phenotype was categorized into three classes: virulent for LC=70–100%, intermediate for LC=10–70%, and avirulent for LC>10%. Three independent repetitions were performed for each isolate.

## Results

### Four *Pm17* events are highly resistant to powdery mildew in three field seasons

**Fig. 1.**
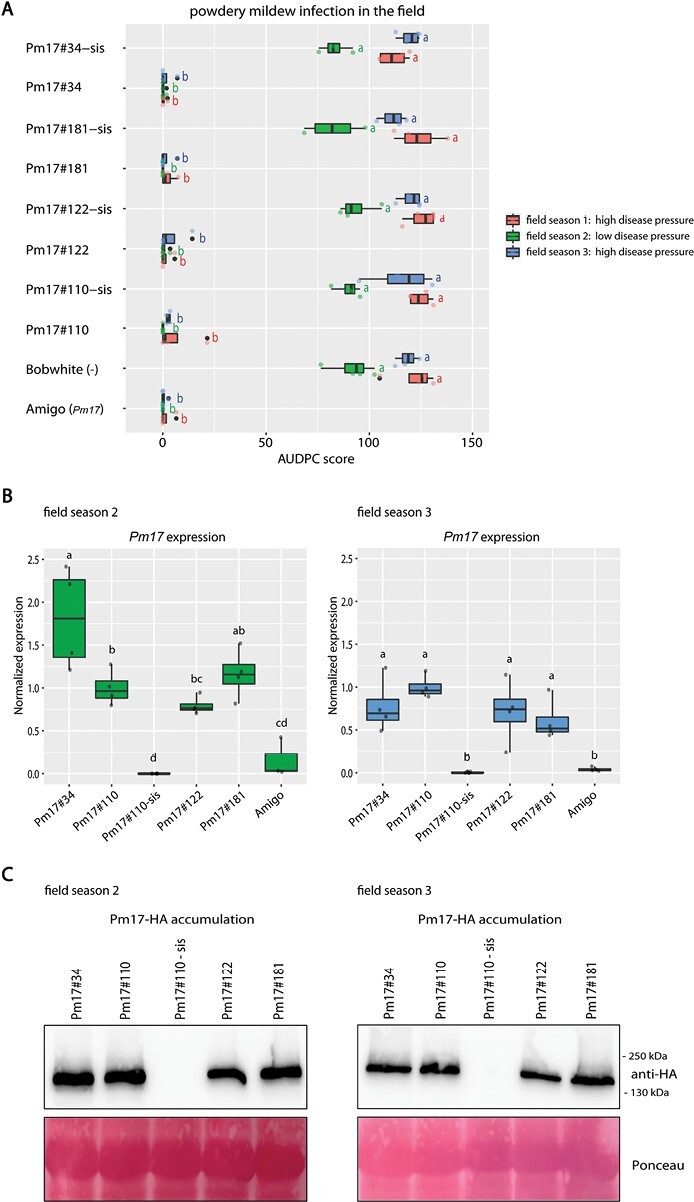
Powdery mildew infection, *Pm17* expression and PM17 protein accumulation in field-grown transgenic *Pm17* Bobwhite events. (A) Powdery mildew infection of field-grown plants. Area under disease progress curve (AUDPC) scores were calculated from four independent plots for each genotype in field seasons 1 (red), 2 (green), and 3 (blue). Non-transformed Bobwhite and wheat cultivar Amigo, which carries endogenous *Pm17*, are included as controls. Powdery mildew disease pressure was high during field seasons 1 and 3, and low during field season 2. Different letters next to the bars denote a significant difference within the same season in the Tukey’s honestly significant difference (HSD) test (α=0.050). (B) *Pm17* expression in flag leaves from field seasons 2 and 3. Expression values were normalized to the expression of reference gene *ADP ribosylation factor* (*ADPRF*) and plotted relative to line Pm17#110. Four biological replicates, each consisting of three pooled flag leaf segments, were used for each genotype in technical duplicates. Different letters above the bars denote a significant difference in expression level (Tukey’s HSD test, α=0.050). (C) PM17–HA protein accumulation in flag leaves from field seasons 2 and 3. Each sample contains three pooled flag leaf segments. Total protein concentration was measured and adjusted to the same concentration prior to loading. Ponceau staining indicates equal loading.

The four *Pm17* overexpression events Pm17#34, Pm17#110, Pm17#122, and Pm17#181 and the corresponding sister lines Pm17#34-sis, Pm17#110-sis, Pm17#122-sis, and Pm17#181-sis were tested for powdery mildew resistance in the field. These events had been generated and tested previously in the laboratory by Singh *et al.* (2018). Sister lines are null segregants of the transgene that do not carry the *Pm17* transgene in the genome but have gone through the same tissue culture procedure. Sister lines are ideal controls to distinguish between pleiotropic effects of the transgene (the sister line does not show the effect) and somaclonal variation (the sister line shows the same effect). The research site where the field trials were performed shows a heavy natural powdery mildew infection every year. To ensure powdery mildew infection in case of an unusual year with little natural infection, we artificially infected the flanking rows of the test plots with Swiss isolate *Bgt* 96224 (Supplementary Fig. S2). During the field seasons, as soon as the powdery mildew infection started, we scored powdery mildew infection every few days for each plot and calculated the area under disease progress curve (AUDPC) score. All four *Pm17* events showed strong powdery mildew resistance (AUDPC score 0–22, median=0) in all three field seasons, whereas the non-transformed Bobwhite and all four sister lines were infected with powdery mildew (AUDPC scores up to 138) (Fig. 1A). The three field seasons differed in disease pressure: whereas field seasons 1 and 3 showed high disease pressure, it was low in field season 2. As an additional control in our trials we included wheat cultivar Amigo, which carries an endogenous *Pm17* under the native *Pm17* promoter. Amigo was powdery mildew resistant in the field (Fig. 1A). Amigo likely carries an additional, yet unidentified powdery mildew resistance gene (Müller *et al.*, 2022) and it is therefore not possible to evaluate with certainty the contribution of natural *Pm17* to the observed resistance. Furthermore, cultivar Amigo is a winter wheat and only a few Amigo plants showed a spring wheat type of growth to allow comparison of mildew infection at similar growth stages.

In field seasons 2 and 3 we collected flag leaf samples of the field-grown plants to measure transgene expression and transprotein accumulation. All four events carry a transgene that encodes a C-terminally HA-tagged PM17. All four *Pm17* events showed high *Pm17* gene expression in both field seasons studied (Fig. 1B). In field season 2, event Pm17#34 showed a significantly higher *Pm17* expression level than events Pm17#110 and Pm17#122. However, in field seasons 3 there was no statistically significant difference in *Pm17* expression levels between the four events (Fig. 1B). *Pm17* expression level in Amigo, which carries endogenous *Pm17* under the native promoter, showed a significantly lower expression level than the transgenic events (Fig. 1B). In both field seasons, all four events showed similar levels of PM17–HA protein accumulation (Fig. 1C).

### PM8 transprotein accumulates in field-grown plants, but provides no powdery mildew resistance

**Fig. 2.**
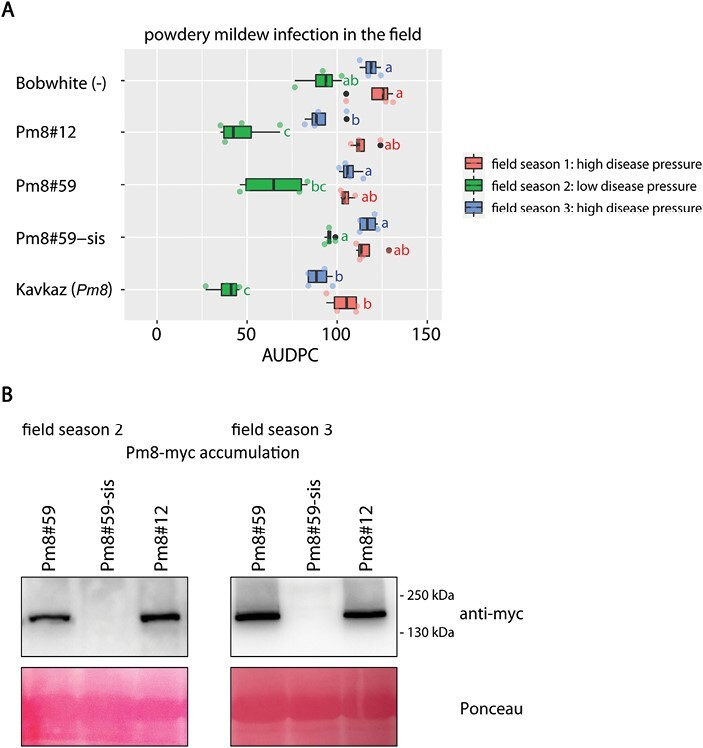
Powdery mildew infection and PM8 protein accumulation in field-grown transgenic *Pm8* Bobwhite events. (A) Powdery mildew infection of field-grown plants. AUDPC scores were calculated from four independent plots for each genotype in field seasons 1 (red), 2 (green), and 3 (blue). Non-transformed Bobwhite, Pm8#59-sis and wheat cultivar Kavkaz, which carries endogenous *Pm8*, are included as controls. Different letters next to the bars denote a significant difference within the same season in Tukey’s HSD test (α=0.050). (B) PM8–myc protein accumulation in flag leaves from field seasons 2 and 3. Each sample contains three pooled flag leaf segments. Total protein concentration was measured and adjusted to the same concentration prior to loading. Ponceau staining indicates equal loading.

We chose the two high *Pm8* expression events, Pm8#12 and Pm8#59, and the corresponding sister line, Pm8#59-sis, to test powdery mildew resistance and plant development in the field. The two events were generated previously and tested in the laboratory by Hurni *et al.* (2013). In field seasons 1 and 3 with high disease pressure, both *Pm8* events were powdery mildew susceptible (Fig. 2A). They showed similar AUDPC scores to non-transformed Bobwhite, sister line Pm8#59-sis, and wheat cultivar Kavkaz, which carries an endogenous *Pm8* under the native *Pm8* promoter (Fig. 2A). In the low disease pressure field season 2, Pm8#12, Pm8#59, and Kavkaz were still powdery mildew infected, but they had lower AUDPC scores than non-transformed Bobwhite and Pm8#59-sis (Fig. 2A). In field seasons 2 and 3 we collected field-grown flag leaf samples to determine transprotein accumulation. The *Pm8* events carry transgenes that encode C-terminally myc-tagged PM8. Both *Pm8* events showed similar levels of PM8–myc transprotein accumulation in both field seasons 2 and 3 (Fig. 2B).

### Combining *Pm17* with *Pm3b*, and *Pm17* with *Pm8* provides additive race-specific powdery mildew resistance in seedling assays

To study compatibility of the overexpressed transgene *Pm17* with the closely related overexpressed transgenes *Pm3b* and *Pm8*, we used an approach of transgene insertion and cross-breeding. Four transgenic Bobwhite events overexpressing non-epitope-tagged *Pm3b* were previously tested in the field for powdery mildew resistance (Brunner *et al.*, 2011). In this study we generated a new transgenic Bobwhite event overexpressing epitope-tagged *Pm3b-HA* under the *ubi* promoter, called event Pm3b#64. We crossed Pm3b#64 with Pm17#110, and during the subsequent generations selected a plant family homozygous for the two transgenes. This homozygous plant family from crossing Pm17#110×Pm3b#64 we simply named Pm17+Pm3b. To generate the second pyramided line, we crossed Pm17#110 with Pm8#59, and during the subsequent generations selected a plant family homozygous for the two transgenes. This homozygous plant family from cross Pm17#110×Pm8#58 we named Pm17+Pm8.

**Fig. 3.**
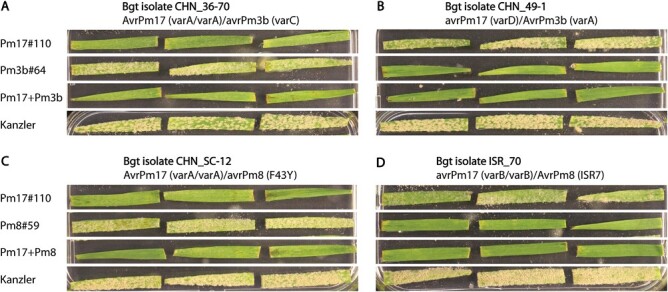
*Bgt* infection tests on pyramided lines Pm17+Pm3b and Pm17+Pm8 and their corresponding parental events. Three biologically independent leaf segments of 10-day-old transgenic Bobwhite wheat seedlings were infected with the indicated *Bgt* isolates. The relevant *Avr* effector gene haplotypes are indicated (Bourras *et al.*, 2019; Müller *et al.*, 2022; Kunz *et al.*, 2023). Wheat cultivar Kanzler was included as a susceptible control. (A) *Pm17* mediated resistance against *Bgt* isolate CHN_36-70 in pyramided line Pm17+Pm3b. (B) *Pm3b* mediated resistance against *Bgt* isolate CHN_49-1 in pyramided line Pm17+Pm3b. (C) *Pm17* mediated resistance against *Bgt* isolate CHN_SC-12 in pyramided line Pm17+Pm8. (D) *Pm8* mediated resistance against *Bgt* isolate ISR_17 in pyramided line Pm17+Pm8.

To test whether the two pyramided transgenes provide additive race-specific powdery mildew resistance, we performed seedling assays under controlled conditions using *Bgt* isolates with a specific genetic makeup of *Avr* effector gene haplotypes. In our *Bgt* isolate collection we identified six isolates with the desired combinations of *Avr* haplotypes. The three *Bgt* isolates CHN_2-5, CHN_36-70, and CHN_SC-12, which were avirulent (or intermediate) on parental event Pm17#110 but virulent on parental event Pm3b#64, showed an avirulent phenotype on Pm17+Pm3b (data of representative isolate CHN_36-70: Fig. 3A). The three *Bgt* isolates CHN_GZ-6, CHN_49-1, and CHN_36-3, which were avirulent (or intermediate) on parental event Pm3b#64 but virulent on parental event Pm17#110, showed an avirulent phenotype on Pm17+Pm3b (data of representative isolate CHN_49-1: Fig. 3B). These results demonstrate an additive race-specific resistance mediated by *Pm17* and *Pm3b* in pyramided line Pm17+Pm3b and the activity of both genes when combined.

Next, we performed the same assay on pyramided line Pm17+Pm8. In our *Bgt* isolate collection we found nine isolates with the desired combinations of *Avr* haplotypes. The five *Bgt* isolates CHN_SD-3, CHN_2-5, CHN_36-70, CHN_SC-12, and CHE_96224 showed an avirulent or intermediate phenotype on parental event Pm17#110 and a virulent phenotype on parental event Pm8#59. On pyramided line Pm17+Pm8, the five isolates showed the same avirulent or intermediate phenotype as on parental event Pm17#110 (data of representative isolate CHN_SC-12: Fig. 3C). Reciprocally, the four *Bgt* isolates ISR_70, ISR_103, USA_7, and USA_85063, avirulent on parental event Pm8#59 but with intermediate virulence on parental event Pm17#110, were avirulent on pyramided line Pm17+Pm8 (data of representative isolate ISR_17: Fig. 3D). This shows that *Pm17* and *Pm8* confer additive powdery mildew resistance when combined in pyramided line Pm17+Pm8.

### Pyramided lines Pm17+Pm3b and Pm17+Pm8 are highly resistant to powdery mildew in three field seasons

**Fig. 4.**
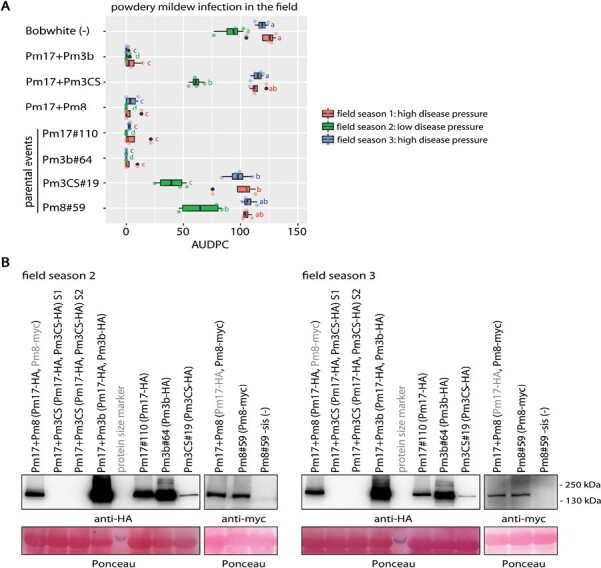
Powdery mildew infection and transprotein accumulation in field-grown transgenic pyramided wheat lines. (A) Powdery mildew infection of field-grown plants. AUDPC scores were calculated from four independent plots for each genotype in field seasons 1 (red), 2 (green), and 3 (blue). Different letters next to the bars denote a significant difference within the same season in Tukey’s HSD test (α=0.050). (B) Transprotein accumulation in flag leaves from field seasons 2 and 3. Each sample contains three pooled flag leaf segments. Total protein concentration was measured and adjusted to the same concentration prior to loading. Ponceau staining indicates equal loading. For pyramided line Pm17+Pm3CS two independent samples (S1 and S2) are included.

We tested the two pyramided lines Pm17+Pm3b and Pm17+Pm8 in the field for powdery mildew resistance. As controls we included parental events Pm17#110, Pm3b#64, and Pm8#59. Pm17+Pm3b and Pm17+Pm8 showed strong powdery mildew resistance during the three field seasons (Fig. 4A). We collected flag leaf samples of the field-grown plants during field seasons 2 and 3 to measure transprotein accumulation. Pyramided line Pm17+Pm3b showed high levels of HA-tagged protein accumulation (PM17–HA and PM3B–HA) during both field seasons. The strong bands in the western blots suggested an additive protein level of PM17–HA and PM3B–HA, comparable to the sum of PM17–HA and PM3B–HA from parental events Pm17#110 and Pm3b#64 (Fig. 4B). Pyramided line Pm17+Pm8 also showed an additive protein level of the two transproteins PM17–HA and PM8–myc: the protein level of PM17–HA from Pm17+Pm18 was similar to the protein level of parental event Pm17#110, and the protein level of PM8–myc from Pm17+Pm8 was similar to the protein level of parental event Pm8#64 (Fig. 4B).

### Transgene promoter silencing in pyramided line Pm17+Pm3CS leads to powdery mildew susceptibility

**Fig. 5.**
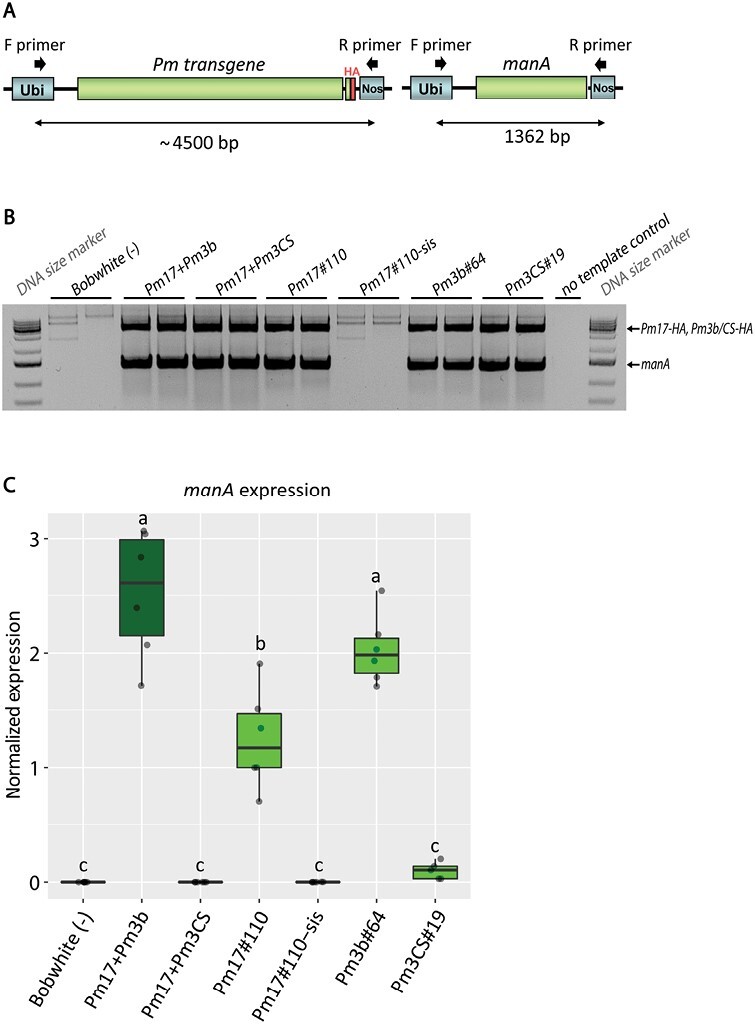
Selection marker gene *manA* genotyping and expression analyses in pyramided lines Pm17+Pm3b and Pm17+Pm3CS. (A) Primer annealing sites in *ubi* promoter and *nos* terminator sequences for genotyping of full-length transgenes. (B) Duplex PCR on genomic DNA using primers shown in (A) for detection of full-length transgenes. (C) RT-qPCR data of *manA* expression in pyramided lines and parental events. Non-transformed Bobwhite and Pm17#110-sis were included as negative controls. Expression values were normalized to expression of reference gene *ADPRF*. Six biological replicates were used for each genotype in technical duplicates. Letters above the bars denote a significant difference in expression level (Tukey’s HSD test, α=0.050).

Previous studies showed that *Pm8*-mediated powdery mildew resistance is suppressed by *Pm3CS* (Hurni *et al.*, 2014). Since the protein sequences of PM17 and PM8 are 82.9% identical, and *Pm17* and *Pm8* are orthologs both originating from rye, we assumed that *Pm17*-mediated resistance could be suppressed by *Pm3CS* as well. *Pm3CS* is a non-functional allele of *Pm3* (Yahiaoui *et al.*, 2006). We expected that, in case of suppression, the non-functional *Pm3CS* together with a suppressed *Pm17* would result in powdery mildew susceptibility. We generated a transgenic Bobwhite event overexpressing epitope-tagged *Pm3CS-HA* under control of the *ubi* promoter, called event Pm3CS#19. We crossed Pm3CS#19 with Pm17#110 and during subsequent generations we selected a plant family homozygous for the two transgenes. The homozygous plant line from cross Pm17#110×Pm3CS#19 we named Pm17+Pm3CS. We grew pyramided lines Pm17+Pm3CS in the field together with parental lines Pm3CS#19 and Pm17#110. Pm17+Pm3CS was powdery mildew susceptible during three field seasons with AUDPC scores similar to non-transformed Bobwhite (Fig. 4A). Parental event Pm3CS#19 was powdery mildew susceptible during the two field seasons 1 and 3 with high disease pressure but showed an intermediate powdery mildew resistance phenotype in field season 2 with low disease pressure (Fig. 4A). We collected flag leaf samples of the field-grown plants during field seasons 2 and 3 to measure transprotein accumulation. Parental event Pm3CS#19 showed a low amount of PM3CS transprotein accumulation, and surprisingly, there was no accumulation of either PM17 or PM3CS in pyramided line Pm17+Pm3CS (Fig. 4B). We measured transgene expression levels in RT-qPCR assays using respective *Pm17-* and *Pm3*-specific primers, and confirmed the results obtained from western blots: there was no transgene expression in pyramided line Pm17+Pm3CS (Supplementary Fig. S3). We confirmed the presence of both full-length error-free transgene sequences *Pm17-HA* and *Pm3CS-HA* in field-grown pyramided line Pm17+Pm3CS by PCR followed by Sanger sequencing. We concluded that both transgenes *Pm17-HA* and *Pm3CS-HA* are present in pyramided line Pm17+Pm3CS, but in contrast to the transgenes in parental lines Pm17#110 and Pm3CS#19, transgenes in pyramided line Pm17+Pm3CS do not produce proteins. We assumed that either the transgenes or the transgene promoters are silenced in pyramided line Pm17+Pm3CS. To test this hypothesis, we took advantage of the presence of the third transgene, the sequence-unrelated selectable marker gene *manA*, in pyramided lines Pm17+Pm3CS and Pm17+Pm3b. *ManA* encodes phosphomannose isomerase, which metabolizes mannose into fructose, a trait used for selection of transformed cells during tissue culture (Wright *et al.*, 2001). *ManA* is expressed under the *ubi* promoter, the same promoter we used for all *Pm* transgenes. Using primers in the *ubi* promoter and *nos* terminator sequences (Fig. 5A) we confirmed the presence of all *Pm* transgenes and *manA* in pyramided lines Pm17+Pm3CS and Pm17+Pm3b, as well as in the corresponding parental events (Fig. 5B). Using RT-qPCR we tested for *manA* expression and found that *manA* was expressed in pyramided line Pm17+Pm3b as well as in parental events Pm17#110 and Pm3b#64 (Fig. 5C). Pyramided line Pm17+Pm3CS did not express *manA* and parental event Pm3CS#19 showed a 12-fold lower *manA* expression level compared with Pm17#110 (Fig. 5C). From these results we concluded that transgenes *Pm17-HA*, *Pm3CS-HA*, as well as *manA* were silenced in pyramided line Pm17+Pm3CS. PM17–HA protein accumulation was much higher in parental event Pm17#110 compared with PM3CS–HA protein accumulation in parental event Pm3CS#19 (Fig. 4B). Thus, there might already be an incomplete transgene silencing in parental event Pm3CS#19, which increased in progeny line Pm17+Pm3CS. We tested *manA* presence and *manA* expression in a second Pm17+Pm3CS plant family from an independent cross of Pm17#110 and Pm3CS#19 where likewise *manA* was not expressed (Supplementary Fig. S4). Thus, a different *Pm3CS* event needs to be used in future crossing experiments. We conclude that *ubi* promoter silencing is the reason for the powdery mildew susceptibility of field-grown Pm17+Pm3CS.

### No pleiotropic or somaclonal variation effects in four field-grown *Pm17* events and pyramided line Pm17+Pm8

**Fig. 6.**
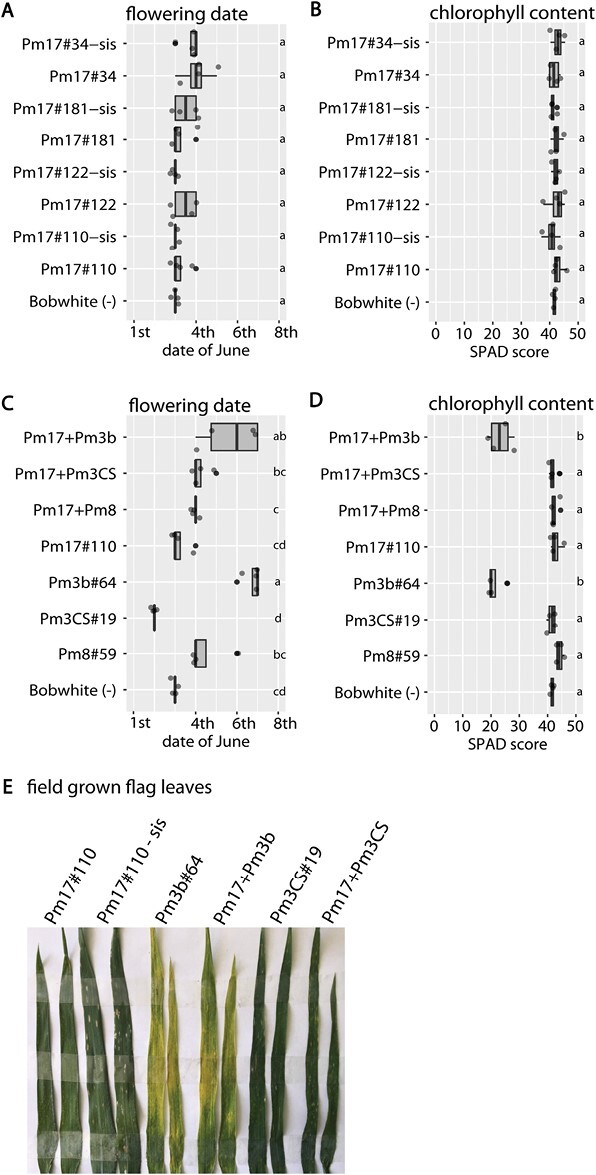
Flowering date, flag leaf chlorophyll content, and photograph of a flag leaf of field-grown transgenic wheat from field season 3. Four plots per genotype were measured (A–D). Different letters denote a significant difference in Tukey’s HSD test (α=0.050). (A) Flowering dates of four *Pm17* events and the corresponding sister lines. (B) Flag leaf chlorophyll content of four *Pm17* events and the corresponding sister lines. SPAD scores of four plants per plot and four plots per genotype were measured using a portable chlorophyll meter. (C) Flowering dates of the pyramided lines and the corresponding parental events. (D) Flag leaf chlorophyll content of the pyramided lines and the corresponding parental events. SPAD scores of four plants per plot and four plots per genotype were measured using a portable chlorophyll meter. (E) Photograph of two representative field-grown flag leaves per indicated genotype.

We tested the field-grown plants for pleiotropic effects of transgene overexpression and for somaclonal variation resulting from tissue culture. No phenotypic variation was observed among the four *Pm17* events, the corresponding sister lines, and the non-transformed Bobwhite. All four *Pm17* events, sister lines, and non-transformed Bobwhite flowered around the same date during all three field seasons (data for field season 3: Fig. 6A) and no statistically significant difference of flag leaf chlorophyll content was measured (data from field season 3: Fig. 6B). However, pyramided line Pm17+Pm3b showed a statistically significant delay of flowering (3 d in field season 3) in each field season compared with non-transformed Bobwhite (data from field season 3: Fig. 6C) and a statistically significant lower flag leaf chlorophyll content (a reduction of 50% of the SPAD score in field season 3) compared with non-transformed Bobwhite (data from field season 3: Fig. 6D). These phenotypes were inherited from parental line Pm3b#64, which showed statistically significant delayed flowering compared with non-transformed Bobwhite (Fig. 6C) and reduced chlorophyll content in flag leaves compared with non-transformed Bobwhite (Fig. 6D). Leaves of field-grown parental event Pm3b#64 and pyramided line Pm17+Pm3b were visibly yellow (Fig. 6E). These phenotypes of Pm3b#64 and Pm17+Pm3b were only visible in the field at adult stage and not in the greenhouse at any stage, which highlights the importance of field trials.

## Discussion

### Several factors contribute to powdery mildew resistance levels in the field

In previous studies with transgenic Bobwhite lines overexpressing single and pyramided *Pm3* alleles, we attributed the observed increase of powdery mildew resistance in the field to two effects, transgene overexpression and *Pm3* allele specificity (Brunner *et al.*, 2011, 2012; Koller *et al.*, 2018, 2019). Resistance strength has also been shown to correlate with expression levels of NLR resistance genes in other plant pathosystems (Feuillet *et al*., 2003; Wang *et al*., 2021). In this study, the four *Pm17* events showed similar levels of *Pm17* expression and PM17 protein accumulation, and high levels of powdery mildew resistance (Fig. 1). We speculate that both high transgene expression and PM17 specificity by recognition of AVRPM17 contributed to the strong powdery mildew resistance phenotype in the field. However, additional *Pm17* events with varying transgene expression levels have to be tested to evaluate the contribution of the expression level to the resistance phenotype. The two *Pm8* events, which both showed similar levels of PM8 protein accumulation in field-grown flag leaves, were powdery mildew susceptible (Fig. 2). Thus, the absence of AVRPM8 recognition likely led to the susceptibility phenotype in the field. Pyramided line Pm17+Pm8 showed the same high level of powdery mildew resistance in the field as parental line Pm17#110 (Fig. 4). Thus, the resistance phenotype of Pm17+Pm8 was mediated by transgene *Pm17* alone, and not by transgene *Pm8*, because parental event Pm8#59 was powdery mildew susceptible. Pyramided line Pm17+Pm3b showed the same high level of powdery mildew resistance in the field as both parental events Pm17#110 and Pm3b#64 (Fig. 4). We speculate that the powdery mildew resistance phenotype of Pm17+Pm3b is more durable than the resistance phenotype of the two parental events due to additive race-specific resistance shown in seedling assays (Fig. 3), but this will need further field testing during more years and at more locations. Since we show that *Bgt* isolates lacking the corresponding AVRs retain full virulence on the specific transgenic events overexpressing *Pm17*, *Pm3b*, or *Pm8* under the *ubi* promoter used in this study (Fig. 3), we exclude that these transgenic events suffer from overexpression artefacts. This retained race-specificity has already been demonstrated previously for overexpressed *Pm17* (Singh *et al*., 2018; Müller *et al*., 2022), *Pm3b* (Brunner *et al*., 2011), and *Pm8* (Hurni *et al*., 2013; Kunz *et al*., 2023). Future studies based on the same genes but under control of the native promoters might reveal the contribution of overexpression versus the effect of gene combinations on improved resistance. Furthermore, virulence analysis of the prevailing *Bgt* isolates during different years and at different locations would be helpful to fully understand effects of overexpression and gene combination.

### Complete transgene promoter silencing in pyramided line Pm17+Pm3CS, but not in parental events Pm17#110 and Pm3CS#19

In pyramided line Pm17+Pm3CS, transproteins PM17 and PM3CS were not observed (Fig. 4B) and transgene *manA* was not expressed (Fig. 5C). While *Pm17-HA* and *Pm3CS-HA* have high sequence homology, *manA* carries no homologous sequences to either *Pm* gene. This suggests that transcriptional gene silencing due to methylation of the common *ubi* promoter is most likely the reason for silencing of all transgenes in pyramided line Pm17+Pm3CS. It is possible that double stranded RNA (dsRNA) containing *ubi* promoter sequences, resulting from inverted repeats that can arise from transgene rearrangements during DNA transformation (Iyer *et al.*, 2000), led to methylation of this promoter. Because the silencing mechanism functions through sequence-specific recognition of the promoter sequences by the dsRNA (Mette *et al.*, 2000), methylation would be established in all copies of the *ubi* promoter. Therefore, all transgenes under the control of this promoter would be silenced. *ubi* promoter silencing has previously also been described in transgenic rice (Kumpatla and Hall, 1998). Field-grown parental event Pm3CS#19 showed low levels of PM3CS protein (Fig. 4B). We assume that this is due to incomplete silencing of the *ubi* promoter, which in progeny line Pm17+Pm3CS progressed to complete *ubi* promoter silencing. In future, promoters less prone for silencing could be used (Schmitz *et al.*, 2022).

### Pleiotropic effects observed in the field but not in the greenhouse emphasize the importance of field trials

We observed chlorotic leaves in field-grown event Pm3b#64 and progeny pyramided line Pm17+Pm3b, but not in any other transgenic genotypes tested in this study (Fig. 6). Greenhouse grown Pm3b#64 and Pm17+Pm3 had no chlorotic leaves, either at seedling or later stages (Fig. 3). Field-grown parental line Pm3b#64 showed higher levels of HA-tagged protein accumulation than the other parental lines tested (Fig. 4B). Pyramided line Pm3b+Pm17 showed the highest levels of HA-tagged protein accumulation from all the tested genotypes. We speculate that overexpression of *Pm3b* in the Bobwhite background can lead to the emergence of pleiotropic effects. This is in accordance with the observations by Brunner *et al.* (2011), where they tested four transgenic *Pm3b* events and the corresponding sister lines in the field, and observed a positive correlation between *Pm3b* expression levels and the emergence of pleiotropic effects. To generate pyramided line Pm17+Pm3b we did not use the *Pm3b* events from Brunner *et al.* (2011), because in those events, the *Pm3b* transgene is not epitope tagged. The difference in plant phenotypes between greenhouse- and field-grown plants confirms the importance of field trials to determine the phenotype in the agricultural environment, where the plants are exposed to a plethora of stimuli, as well as biotic and abiotic stresses. It is still complex and laborious in Switzerland to obtain permission for field trials with transgenic crops and to run these trials, and the field trials are potentially still threatened by vandalism (Romeis *et al.*, 2013; Brunner *et al.*, 2021). However, these obstacles should not prevent researchers from performing field trials, which are essential to test transgene function and possible pleiotropic effects.

### Considerations on the future of using a transgenic approach for pyramiding of resistance genes from the extended gene pool of wheat

Our work shows, on the example of pyramided line Pm17+Pm3b, the potential of combining overexpressed NLR-encoding genes from the extended gene pool of wheat to provide strong disease resistance in the field. However, several aspects need further optimization. To thoroughly test a transgene, many events with varying levels of transgene expression need to be tested in the field, together with the corresponding sister lines. Brabham *et al.* (2023, Preprint) showed that high expression levels of single transgenic NLRs can lead to higher resistance against wheat stem rust. Fine tuning of transgene expression level is crucial to optimize resistance while minimizing possible pleiotropic effects leading to fitness costs for the plants. A study performed with transgenic maize and soybean found that transgene expression levels were mainly impacted by the choice of promoter and the choice of genetic background cultivar, while the genomic site of transgene insertion played a minor role (Betts *et al.*, 2019). Recent technological progress enables the transformation of any wheat genotype of interest (Debernardi *et al.*, 2020; Wang *et al.*, 2022; Johnson *et al.*, 2023), which greatly facilitates the testing of transgenes in different genetic backgrounds. In this study we used a combined approach of transgene insertion by biolistic transformation and subsequent cross-breeding to generate plants overexpressing two pyramided *NLR* type of resistance genes from the primary and the tertiary gene pool of wheat. In another study Luo *et al.* (2021) achieved high field resistance against wheat stem rust by pyramiding of five resistance transgenes in wheat cultivar Fielder. They used *Agrobacterium*-mediated transformation for the integration of a large transgene cassette containing all five resistance genes. Inserting DNA fragments into plant genomes by biolistic- or *Agrobacterium*-mediated transformation has so far not been well accepted politically, especially not in Europe (Wulff and Dhugga, 2018). In the future, the more politically accepted genome editing technology could be used (Dima *et al.*, 2022; FAO, 2022). So far insertion of large DNA fragments by genome editing has been challenging in plants, but recently an optimized approach called PrimeRoot was introduced (Sun *et al.*, 2023; Wang and Doudna, 2023). Together, the above-mentioned approaches will facilitate the deployment of the existing *NLR* gene diversity (Barragan and Weigel, 2021) from the extended gene pool of wheat, by inserting *NLR* genes with additive resistance effects quickly and precisely into wheat cultivars of interest, to achieve strong and durable disease resistance.

## Supplementary data

The following supplementary data are available at *JXB* online.

Fig. S1. *Pm8* and *Pm17* introgression from rye into wheat compared with transgenic *Pm8* and *Pm17* events in wheat.

Fig. S2. Infection test of transgenic Bobwhite wheat with powdery mildew isolate *Bgt* 96224.

Fig. S3. *Pm17* and *Pm3* expression of field-grown transgenic pyramided lines Pm17+Pm3b and Pm17+Pm3CS, and their parental events determined by RT-qPCR.

Fig. S4. Selectable marker gene *manA* expression analysis in seedlings of four plant families of pyramided line Pm17+Pm3CS using RT-qPCR.

erad493_suppl_Supplementary_Figures_S1-S4

## Data Availability

All data supporting the findings of this study are included in the paper and its supplementary data, or are available from the corresponding author on request.
