# Supplementary material for: Pyramiding of transgenic immune receptors from primary and tertiary wheat gene pools improves powdery mildew resistance in the field
Source: J Exp Bot. 2023 Dec 10;75(7):1872–86. doi: 10.1093/jxb/erad493 (PMC10967238; doi:10.1093/jxb/erad493)
Supplement: erad493_suppl_Supplementary_Figures_S1-S4 [file erad493_suppl_supplementary_figures_s1-s4.pdf]

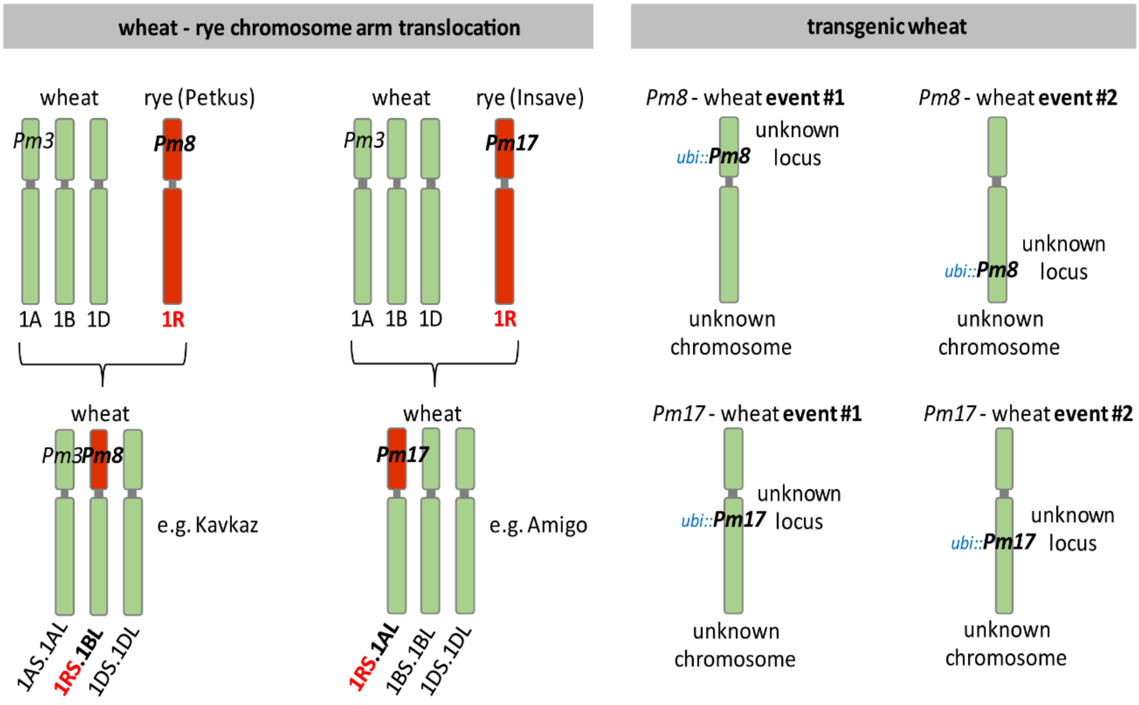

Supplementary Figure S1. *Pm8* and *Pm17* introgression from rye into wheat compared to transgenic *Pm8* and *Pm17* events in wheat. *Pm8* is localized on 1RS.1BL in wheat and *Pm17* is localized on 1RS.1AL, respectively. Transgenic *Pm17* and *Pm8* wheat events generated by biolistic transformation carry the transgenes at unknown loci in the genome.

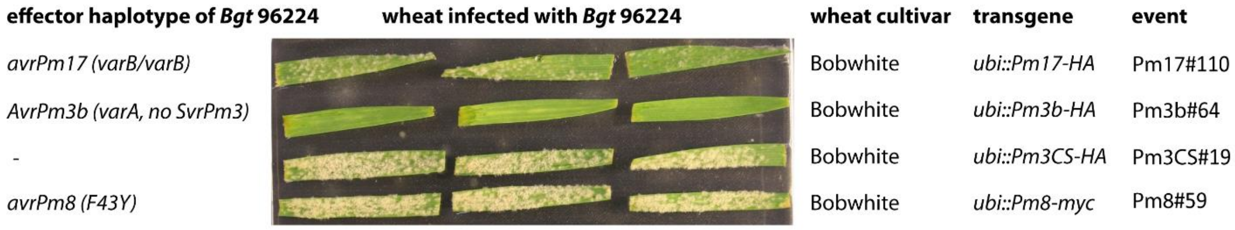

Supplementary Figure S2. Infection test of transgenic Bobwhite wheat with powdery mildew isolate *Bgt* 96224. Three biologically independent leaf segments of ten-day-old transgenic Bobwhite wheat seedlings expressing either *Pm17, Pm3b, Pm3CS* or *Pm8* under the *ubi* promoter were infected with *Bgt* 96224. The relevant *Avr* effector gene haplotype of *Bgt* 96224 is indicated.

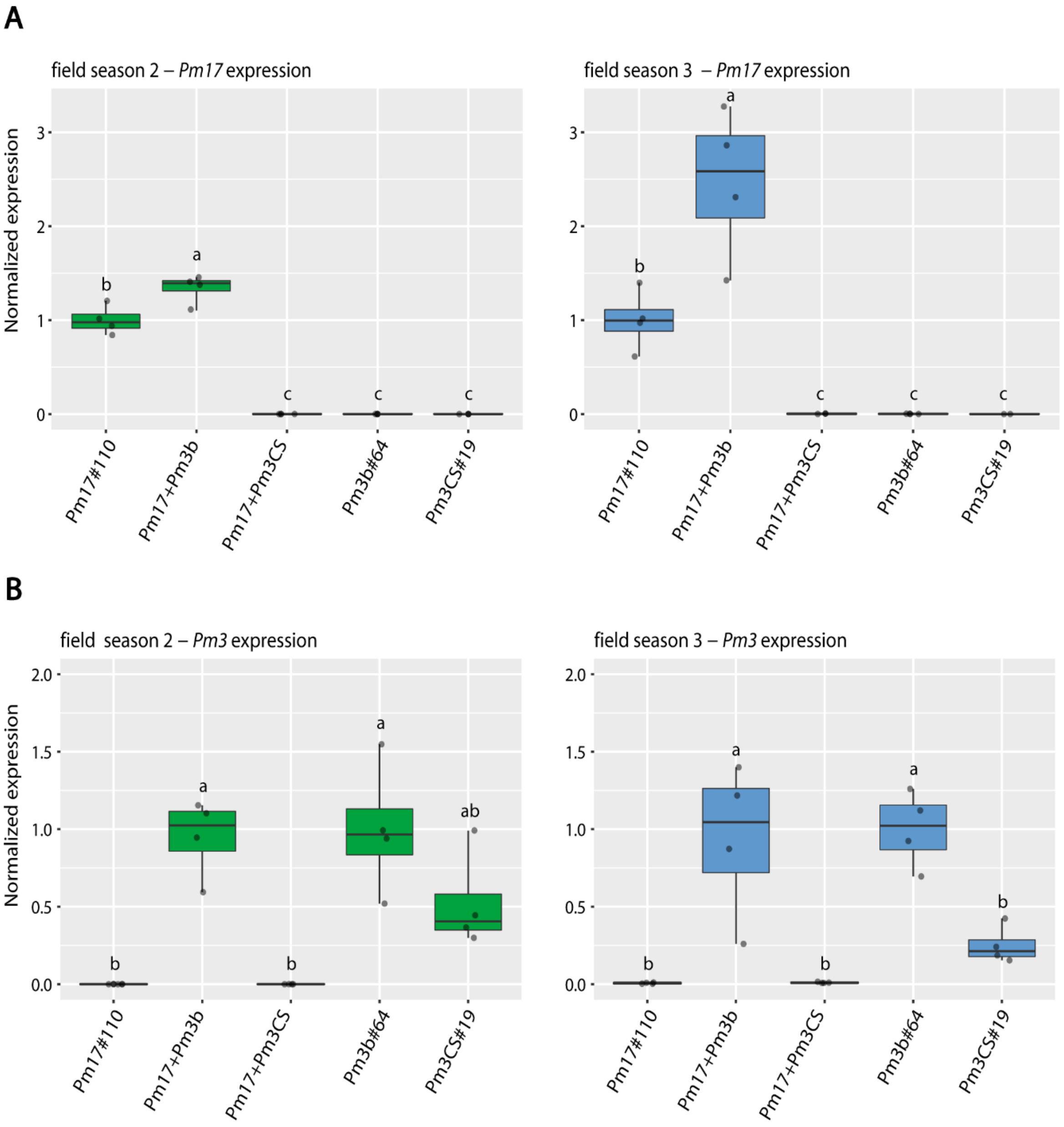

Supplementary Figure S3. *Pm17* (A) and *Pm3* (B) expression of field grown transgenic pyramided lines Pm17+Pm3b and Pm17+Pm3CS, and their parental events determined by RT-qPCR. Expression values were normalized to expression of reference gene *ADPRF* and plotted relative to line Pm17#110 (A) and line Pm3b#64 (B). Four biological replicates, each consisting of three pooled flag leaf segments, were used for each genotype in technical duplicates. Letters on top of the bars denote a significant difference in expression level (TukeyHSD test, α=0.050).

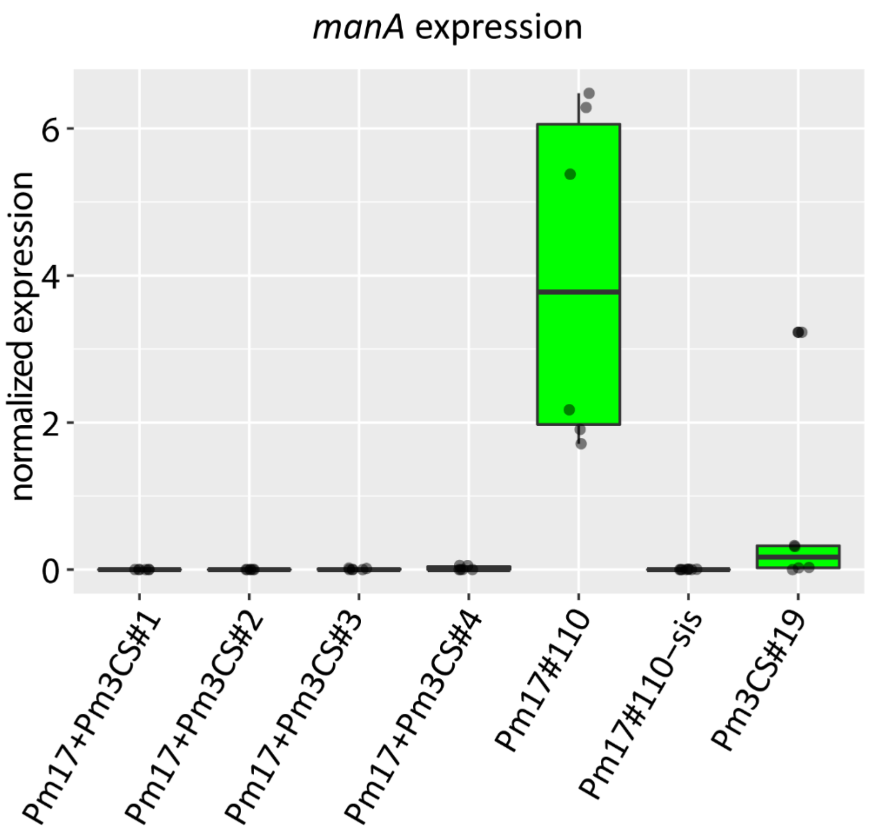

Supplementary Figure S4. Selectable marker gene *manA* expression analysis in seedlings of four plant families of pyramided line Pm17+Pm3CS using RT-qPCR. Plant families #1 and #2 originate from the same F0 spike B. Plant families #3 and #4 both originate from the same F0 spike D. Spike B and spike D represent independent crosses of parental events Pm17#110 with Pm3CS#19. Expression values were normalized to expression of reference gene *ADPRF*. Six biological replicates were used for each genotype in technical duplicates.
